# Finite Element Analysis of Low-Profile Reconstruction Plates for Atrophic Mandibles—Part II: A Comparison of Customized Plates with 3D Grid-Type and Conventional Designs

**DOI:** 10.3390/cmtr19010009

**Published:** 2026-01-23

**Authors:** Bianca Pulino, Robert Sader, Guilherme Louzada, Majeed Rana, Gabriele Millesi, Geraldo Prestes de Camargo Filho, Raphael Capelli Guerra

**Affiliations:** 1 Hospital Sírio-Libanês, Instituto de Ensino e Pesquisa, São Paulo 01308-050, Brazil; 2 Faculdade Israelita de Ciências da Sáude Albert Einstein, Department of Oral and Maxillofacial Surgery, São Paulo 05652-900, Brazil; 3 Hospital Israelita Albert Einstein, Department of Oral and Maxillofacial Surgery, São Paulo 05652-900, Brazil; 4 Department of Craniomaxillofacial, Plastic and Reconstruction Surgery, Goëthe Universität Frankfurt, 60325 Frankfurt, Germany; 5 University Hospital Vienna, Department of Oral and Maxillofacial Surgery, 1090 Vienna, Austria; 6 Dental School, Nova Southeastern University, Clearwater, FL 33759, USA

**Keywords:** mandible, mandibular fixation technique, edentulous mandible, finite element analysis, mandibular reconstructions

## Abstract

Objectives: The aim of this study was to compare the stiffness-related mechanical response and peak von Mises stress distribution of low-profile 2.4 mm mandibular reconstruction systems (a conventional reconstruction plate, a 3D grid-type plate, and a customized plate) in a virtual atrophic mandible model with a 5 cm segmental defect. Materials and Methods: A CT-based three-dimensional mandible model was created and instrumented with three plate configurations (G1–G3). Linear static finite element analyses were performed under a 300-N masticatory load combined with literature-based muscle force vectors. Peak von Mises stresses were recorded for plates and screws, and the locations of maximum stress concentration were identified. Results: Peak plate stress was highest in the conventional reconstruction plate (G1: 695.5 MPa), followed by the 3D grid-type plate (G2: 595.6 MPa), and lowest in the customized plate (G3: 185.2 MPa). The peak screw stress was 692.9 MPa (G1), 898.0 MPa (G2), and 595.6 MPa (G3). The 3D grid-type plate increased construct stiffness but shifted stress concentration toward the mandibular angle and adjacent screws, whereas the customized plate reduced the peak plate stress and limited the extent of the high-stress region across the defect. Conclusions: Within the limitations of a linear static FEA (stiffness/stress distribution rather than failure load or fatigue resistance), the customized plate (G3) demonstrated the most favorable biomechanical performance (lowest peak plate stress). The 3D grid-type plate (G2) reduced peak plate stress compared with the conventional design (G1) but produced the highest peak screw stress. Practical considerations such as manufacturing lead time and resource requirements may favor off-the-shelf plates; however, a formal cost or operative-time analysis was not performed.

## 1. Introduction

Individuals with atrophic mandibles often present reduced bone volume and compromised vascularity, which can limit osteogenesis and impair healing. This condition is frequently observed in older, edentulous patients and may be associated with nutritional limitations, impaired oral function, and reduced quality of life. Mandibular defects due to benign or malignant lesions, trauma, or infection can further result in major functional deficits (speech, mastication, swallowing, and airway support) and increase the burden of care through prolonged rehabilitation and the need for secondary interventions [1,2,3].

Reconstruction with load-bearing plates, with or without vascularized or non-vascularized grafts, remains an established strategy; however, postoperative complications such as screw loosening, plate fracture, and infection continue to be reported, with occurrence rates ranging from 28% to 39% [4]. Plate geometry (width, thickness, and hole/screw distribution) influences construct stiffness and how loads are transferred to screws and bone, particularly in long-span segmental defects. In current practice, plate selection is often guided by availability and surgeon experience, because direct in vivo measurement of internal stresses is not feasible and experimental testing can be limited by specimen variability and simplified loading conditions.

Conventional evaluation of fixation constructs relies on clinical case series and in vitro testing using cadaveric or synthetic models. While these approaches provide important insights, they are typically constrained in reproducing patient-specific anatomy, bone quality, and complex load transfer across plate–screw–bone interfaces. As a result, there remains a need for complementary computational methods capable of comparing construct mechanics under standardized conditions and identifying regions prone to stress concentration in anatomically realistic reconstructions.

Low-profile 2.4 mm systems may reduce hardware prominence and facilitate soft-tissue management, but their reduced cross-sectional height can decrease bending stiffness. Consequently, design-related strategies (e.g., increased plate width, 3D grid-type architectures, or patient-specific customization) may be biomechanically relevant for large defects in atrophic mandibles.

Finite element analysis (FEA) is a technique that divides a domain into smaller areas to allow for mathematical modeling of structures and the application of forces at any point [5]. In simple terms, FEA enables the estimation of internal stresses and load paths that cannot be measured easily in real patients, supporting a non-destructive comparison of fixation strategies in anatomically complex regions such as the mandible [6]. Prior FE studies have explored mandibular reconstruction plate–screw–bone mechanics and identified critical regions of stress concentration near the defect and around the first screw on the loaded side (“crucial screw”) [7,8,9,10,11,12,13,14]. Nevertheless, comparative evidence remains limited for low-profile 2.4 mm systems, particularly when contrasting conventional reconstruction plates with newer 3D grid-type and customized designs in atrophic mandibles with large segmental defects.

Therefore, the aim of this study was to compare the stiffness-related mechanical response and stress distribution of three low-profile 2.4 mm plate systems—conventional reconstruction (G1), 3D grid-type (G2), and customized (G3)—in a virtual atrophic mandible with a 5 cm segmental defect without bone grafting. We hypothesized that the customized plate (G3) would reduce peak von Mises stresses in the plate–screw assembly compared with G1 and G2, and that the 3D grid-type plate (G2) would increase construct stiffness and potentially shift peak stresses toward the mandibular angle and adjacent screws. The primary outcomes were the peak von Mises stresses on plates and screws and the locations of maximum stress concentration, with secondary assessment of stiffness-related response via displacement under the applied load.

## 2. Materials and Methods

### 2.1. Study Design and Model Generation

This in silico study compared three low-profile 2.4 mm mandibular reconstruction plate designs applied to an atrophic mandible with a 5 cm segmental defect (n = 1 virtual model per configuration: G1–G3).

A three-dimensional mandible model was generated from computed tomography (CT) images of a patient who underwent mandibular resection (axial slices, 1 mm interval). CT segmentation and surface reconstruction were performed in *Dolphin Imaging Software/32*, Chatsworth, CA 91311, USA, Dolphin Imaging. (Version: 11.9, 11.7 e 9.0), and the surface model was exported in STL format for further processing.

### 2.2. Ethical Approval

This study was authorized through a waiver of informed consent and approved by the Research Ethics Committee involving human subjects via Plataforma Brasil (CAAE: 80036024.4.0000.5420; approval date: 22 May 2024).

### 2.3. Implant Modeling

Plates were modeled in Geomagic Freeform Plus (3D Systems, Rock Hill, SC, USA) and positioned to match the external contour of the mandible, spanning the defect. Three configurations were analyzed:

- G1: Conventional low-profile 2.4 mm reconstruction plate (135 × 8 × 2.4 mm) with bicortical fixation.
- G2: A 3D grid-type low-profile 2.4 mm plate (150 × 17 × 2.4 mm) with bicortical fixation.
- G3: Customized low-profile 2.4 mm plate (140 × 22 × 2.4 mm) with bicortical fixation.

Screws were modeled with a 2.4 mm diameter; the screw closest to the defect was positioned 10 mm from the resection margin.

**Figure 1:**
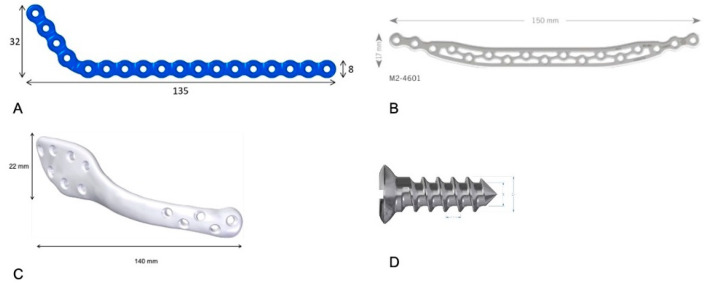
Plate and screw geometries used for finite element modeling. (**A**) 3D grid-type plate: 150 × 17 × 2.4 mm. (**B**) Conventional low-profile 2.4 mm reconstruction plate: 135 × 8 × 2.4 mm. (**C**) Customized low-profile 2.4 mm plate: 140 × 22 × 2.4 mm. (**D**) Bicortical screw: 2.4 mm diameter (length as modeled).

The Figure 1 shows the mandibular plates and the type of screw used in this study.

**Figure 2:**
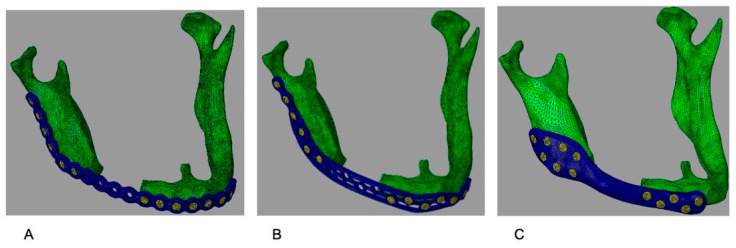
Virtual atrophic mandible models with a 5 cm segmental defect (no bone graft) and the three fixation configurations. (**A**) G1: conventional reconstruction plate. (**B**) G2: 3D grid-type plate. (**C**) G3: customized plate.

Figure 2 illustrates the plates with the correct adaptation in the atrophic mandible.

### 2.4. Material Properties

All materials were modeled as elastic, isotropic, and linearly elastic. The Young’s modulus (E) and Poisson’s ratio (ν) were adopted from prior finite element studies on mandibular reconstruction systems [9,11,15]. The values used in the simulations are summarized in Table 1.

### 2.5. Bone Representation

To focus on comparative construct behavior, the mandible was modeled as a homogeneous isotropic material representing averaged properties of an atrophic mandible (Table 1). This assumption is addressed as a limitation.

### 2.6. Mesh Generation and Element Type

The assembly (mandible, plate, and screws) was discretized with linear tetrahedral solid elements. A mesh convergence study was conducted by refining the mesh until changes in peak von Mises stress were <5%. Final mesh characteristics for each model are reported in Table 2.

### 2.7. Boundary Conditions and Contact Definition

To prevent rigid body motion, both condyles were constrained in all degrees of freedom. Plate–bone interaction was defined as surface-to-surface contact, and screw–bone and screw–plate interfaces were defined as rigid coupling constraints to represent bicortical fixation (the thread geometry is not explicitly modeled).

### 2.8. Loading Conditions

A 300 N load was applied perpendicular to the horizontal plane of the mandible to represent masticatory loading. In addition, muscle forces from the masseter, temporalis, medial pterygoid, and anterior belly of the digastric were applied as vector loads, with directions/origins and magnitudes taken from the literature [15].

### 2.9. Solver and Output Measures

Linear static analyses were run in Ansys (Ansys 2024 R1 24.1) for each configuration. The prespecified outcomes were (i) maximum von Mises stress (MPa) on the plate, (ii) maximum von Mises stress (MPa) on the screws, and (iii) the anatomical/construct locations of these maxima. As a stiffness-related indicator, the displacement at the load application point was also recorded to enable standardized comparison of load transfer behavior across designs. These outputs were selected because regions of elevated plate or screw stress may represent mechanically vulnerable sites and can support preoperative construct selection when planning fixation in long-span atrophic mandibular defects.

### 2.10. Software Reporting

Dolphin Imaging Software/32 da Dolphin Imaging. (Versões: 11.9, 11.7 e 9.0); Geomagic Freeform Plus 3.0 (3D Systems); and Ansys (Ansys 2024 R1 24.1); Abaqus/CAE 2019 software were used.

## 3. Results

### 3.1. Peak Stress on Plates

**Figure 3:**
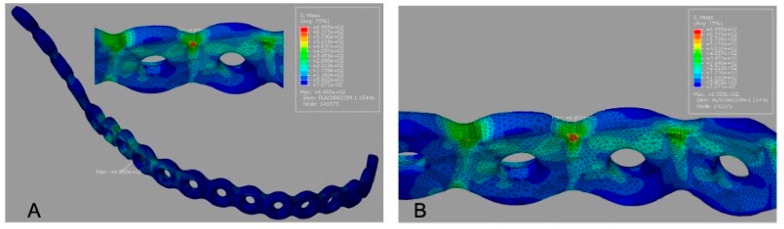
Von Mises stress distribution for G1 (conventional reconstruction plate). (**A**) Plate stress map (MPa; color bar shown; peak 695.5 MPa) with maximum concentration across the segmental defect span. (**B**) Screw stress map (MPa; color bar shown; peak 692.9 MPa) within the plate–screw–bone assembly, highlighting the region adjacent to the defect.

Von Mises stress maps for all models are presented with color scale bars (MPa). The conventional reconstruction plate (G1) showed the highest peak plate stress (695.5 MPa), concentrated along the plate span over the segmental defect (Figure 3A).

**Figure 4:**
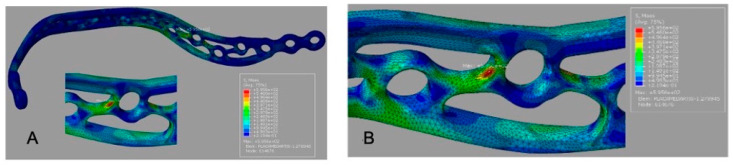
Von Mises stress distribution for G2 (3D grid-type plate). (**A**) Plate stress map (MPa; color bar shown; peak 595.6 MPa) with maximum concentration in the mandibular angle region. (**B**) Screw stress map (MPa; color bar shown; peak 898.0 MPa) within the assembly, indicating higher screw loading near the mandibular angle/loaded side.

The 3D grid-type plate (G2) exhibited a lower peak plate stress (595.6 MPa) compared with G1, with the maximum concentrated in the mandibular angle region near the load application (Figure 4A).

**Figure 5:**
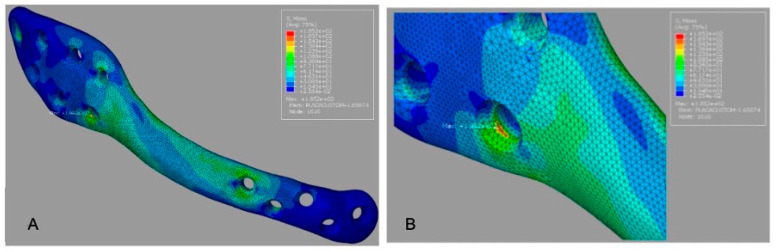
Von Mises stress distribution for G3 (customized plate). (**A**) Plate stress map (MPa; color bar shown; peak 185.2 MPa) demonstrating a reduced high-stress region. (**B**) Screw stress map (MPa; color bar shown; reported peak 595.6 MPa) within the assembly.

The customized plate (G3) demonstrated the lowest peak plate stress (185.2 MPa) and a smaller high-stress region than the other designs (Figure 5A).

### 3.2. Peak Stress on Screws

Peak screw stress values are summarized in Table 3. For G1, the peak screw stress was 692.9 MPa (Figure 3B). For G2, the peak screw stress increased to 898.0 MPa (Figure 4B), indicating a transfer of load toward the screw–bone interface in the mandibular angle region. For G3, the reported peak screw stress was 595.6 MPa (Figure 5B); this value should be rechecked against the solver output because it equals the reported peak plate stress for G2.

### 3.3. Summary Comparison

Overall, the customized plate (G3) produced the lowest peak plate stress and a more diffuse stress distribution across the construct. The 3D grid-type plate (G2) reduced peak plate stress relative to the conventional design (G1) but resulted in the highest peak screw stress. These patterns suggest distinct load-sharing behavior among designs, which may be clinically relevant when selecting fixation strategies for long-span atrophic mandibular defects where screw overload or plate overloading is a concern.

## 4. Discussion

This study used finite element analysis (FEA) to compare three low-profile fixation designs under identical boundary and loading conditions. FEA is suitable in this context because it can simulate internal stress and load transfer within plate–screw–bone assemblies that cannot be measured directly in vivo, while controlling for anatomical and loading variability. Importantly, the present analysis evaluates stiffness-related behavior and stress concentration patterns under linear static loading; it does not estimate failure load or fatigue performance.

Across configurations, the customized plate (G3) exhibited the lowest peak plate stress and a more distributed stress field, consistent with a geometry that increases effective load-bearing area and reduces local stress raisers. The 3D grid-type plate (G2) reduced peak plate stress relative to the conventional plate (G1), suggesting improved stress distribution across the defect span; however, G2 also showed higher peak screw stress, indicating a potential trade-off whereby plate stiffness and geometry shift a greater proportion of load to fixation points near the mandibular angle.

These findings align with prior biomechanical and FE studies reporting a stress concentration near the first screw adjacent to the defect and around the mandibular angle in long-span reconstructions [7,8,9,10,11,12,13]. The present work extends this literature by directly comparing a conventional low-profile plate with a 3D grid-type design and a customized plate in the same atrophic mandible geometry and defect length, highlighting how design-driven load paths can reduce plate stress at the expense of increased screw stress in specific regions.

From a treatment-planning perspective, the prespecified outcomes (peak plate stress, peak screw stress, and their locations) may help surgeons anticipate mechanically vulnerable regions and select fixation strategies accordingly [16,17]. For example, designs that reduce plate stress concentration (as observed for G3) may be preferable when plate overloading is a concern in long defects or poor bone stock, whereas designs associated with higher screw stress (as observed for G2) may warrant attention to screw number, position, and bone quality at the mandibular angle region. These biomechanical inferences should be interpreted cautiously and require experimental validation and clinical follow-up before being translated into definitive clinical recommendations.

Patient-specific plates may require additional steps (imaging processing, design, manufacturing, and logistics) that can influence workflow. In contrast, off-the-shelf 3D grid-type plates may offer a practical alternative when customization is not feasible. Because operative time and costs were not measured, these factors are presented as qualitative considerations rather than study outcomes [18,19,20]

The strengths of this study include the use of a CT-based three-dimensional mandible model, standardized comparison across three clinically relevant low-profile designs, and transparent reporting of material properties, contacts, boundary conditions, and mesh convergence to support reproducibility.

Limitations should be considered when interpreting absolute stress magnitudes. The simulations used a single CT-based mandible model and a linear elastic, static loading framework; therefore, stress values may differ in vivo due to inter-patient variation in anatomy and bone quality, non-linear contact behavior, and time-dependent effects [21]. The mandible was modeled as homogeneous bone and soft tissues were not included; muscle forces and dynamic mastication patterns were simplified to a representative loading scenario. In addition, thread-level screw–bone mechanics and fatigue or failure behavior were not modeled. Despite these constraints, the standardized setup supports relative, between-design comparisons and helps generate hypotheses regarding load-sharing mechanisms that can be tested in future experimental and clinical studies.

## 5. Conclusions

This finite element study compared three low-profile 2.4 mm mandibular reconstruction constructs (conventional reconstruction plate, 3D grid-type plate, and customized plate) in an atrophic mandible with a 5 cm segmental defect, focusing on stiffness-related behavior and peak stress concentrations on plates and screws.

Biomechanically, the customized plate (G3) demonstrated the most favorable stress profile, with the lowest peak plate stress and a more distributed stress field. The 3D grid-type plate (G2) reduced peak plate stress relative to the conventional reconstruction plate (G1) but generated higher peak screw stresses, underscoring a trade-off between plate stress reduction and potential screw overloading in specific regions.

Clinically, these findings may support construct selection for long-span atrophic mandibular defects by highlighting where stress concentrations are likely to occur. In scenarios where minimizing plate stress concentration is prioritized (e.g., extensive defects, limited bone stock, or concern for plate overloading), a customized design may be considered. When customization is not feasible, a 3D grid-type plate may represent a practical alternative, with attention to screw placement and bone quality near the mandibular angle to mitigate localized screw stress. These recommendations should be interpreted as biomechanical guidance and require experimental and clinical validation.

## Figures and Tables

**Table 1:** Material properties used in the simulations.

Model	Element Type	No. of Elements	No. of Nodes	Convergence Criterion
G1—Conventional reconstruction plate	Linear tetrahedral solid element	≈92,000	≈16,000	Δ peak von Mises stress < 5%
G2—3D grid-type plate	Linear tetrahedral solid element	≈92,000	≈16,000	Δ peak von Mises stress < 5%
G3—customized plate	Linear tetrahedral solid element	≈92,000	≈16,000	Δ peak von Mises stress < 5%

**Table 2:** Mesh characteristics and convergence criteria.

Material/Component	Young’s Modulod, E (GPa)	Poisson’s Ratio, υ	Source
Mandible (atrophic bone; homogeneous isotropic)	13.7	0.30	Adopted from previous FEA studies [9,11,15]
Titanium alloy (plates and screws)	110	0.34	Adopted from previous FEA studies [9,11,15]

**Table 3:** Peak von Mises stresses on plates and screws and locations of maximum stresses.

Model	Peak Plate Stress (MPa)	Peak Screw Stress (MPa)	Location of Peak Plate Stress	Location of Peak Screw Stress
G1—Conventional reconstruction plate	695.5	692.9	Plate span over the segmental defect	Screw adjacent to the defect on the loaded side (distal end)
G2—3D grid-type plate	595.6	898.0	Mandibular angle region near load application	Screws in the mandibular angle/loaded side region
G3—Customized plate	185.2	595.6	Localized region along the plate spanning the defect (reduced high-stress area) Peak screw stress for G3 should be verified because it matches the reported peak plate stress for G2.	Screw adjacent to the defect on the loaded side

## Data Availability

The original contributions presented in this study are included in the article. Further inquiries can be directed to the corresponding author(s).
